# Accelerated and Improved Quantification of Lymphocytic Choriomeningitis Virus (LCMV) Titers by Flow Cytometry

**DOI:** 10.1371/journal.pone.0037337

**Published:** 2012-05-17

**Authors:** Darlynn Korns Johnson, Dirk Homann

**Affiliations:** 1 Integrated Department of Immunology, University of Colorado Denver and National Jewish Health, Denver, Colorado, United States of America; 2 Department of Anesthesiology, University of Colorado Denver, Aurora, Colorado, United States of America; University of Iowa, United States of America

## Abstract

Lymphocytic choriomeningitis virus (LCMV), a natural murine pathogen, is a member of the Arenavirus family, may cause atypical meningitis in humans, and has been utilized extensively as a model pathogen for the study of virus-induced disease and immune responses. Historically, viral titers have been quantified by a standard plaque assay, but for non-cytopathic viruses including LCMV this requires lengthy incubation, so results cannot be obtained rapidly. Additionally, due to specific technical constraints of the plaque assay including the visual detection format, it has an element of subjectivity along with limited sensitivity. In this study, we describe the development of a FACS-based assay that utilizes detection of LCMV nucleoprotein (NP) expression in infected cells to determine viral titers, and that exhibits several advantages over the standard plaque assay. We show that the LCMV-NP FACS assay is an objective and reproducible detection method that requires smaller sample volumes, exhibits a ∼20-fold increase in sensitivity to and produces results three times faster than the plaque assay. Importantly, when applied to models of acute and chronic LCMV infection, the LCMV-NP FACS assay revealed the presence of infectious virus in samples that were determined to be negative by plaque assay. Therefore, this technique represents an accelerated, enhanced and objective alternative method for detection of infectious LCMV that is amenable to adaptation for other viral infections as well as high throughput diagnostic platforms.

## Introduction

Lymphocytic choriomeningitis virus (LCMV), an enveloped bi-segmented RNA virus and natural murine pathogen, is the prototypic member of *Arenaviridae*, a family that also includes human pathogenic viruses such as Lassa, Junin, Machupo and Whitewater Arroyo that can cause severe viral hemorrhagic fevers in humans. LCMV infects a wide variety of mammalian cells and has been extensively studied as a model for acute and chronic viral disease, virus-specific immunity and host-pathogen interactions in general [1], [2]. Components of the LCM virion that constitute suitable targets for the quantification of viral infection include the ambisense RNA genome, surface glycoproteins (GP1 and GP2) and the viral nucleoprotein (NP), which forms a two dimensional lattice with the viral RNA inside the viral envelope [2]. The LCMV-NP, in particular, is an attractive target because NP is essential for viral propagation and intracellular NP levels increase during the course of active infection [3], [4].

Historically, quantification of LCMV in tissue samples and virus stocks has been determined using a modified version of plaque assays originally developed for rubella and poliovirus [5], [6], [7], [8]. Since viral titers are determined by visually counting plaques that develop as a result of viral destruction of a cell monolayer, this method requires prolonged incubation of virus with target cells, especially for non-cytopathic viruses such as LCMV. The sensitivity of the plaque assay is limited by the surface area of the tissue culture plates utilized, the large assay volumes utilized (up to 500 µl) and that often require 100-fold sample dilutions for analysis, and the ability of the human eye to discern individual plaques, which in the case of LCMV are particularly heterogeneous.

Due to constraints dictated by the extended incubation time required, observer subjectivity, and limited sensitivity of the plaque assay, there is a need for more expedient, sensitive methods for viral detection. Several promising techniques, including Real-Time RT-PCR for GP and NP transcripts, have been developed and utilized with some frequency for *in vivo* and *in vitro* detection of LCMV. Quantitation of LCMV RNA is highly reproducible and sensitive, detecting as few as five RNA copies or the equivalent of 10 PFU/ml of virus [9], [10], [11]. However, one potential drawback of quantification of viral titers in tissues and serum by Real-time RT-PCR is that the number of viral RNA copies present cannot be directly correlated with infectious virus, particularly in light of the well-characterized presence of defective interfering virions in LCMV infection [12], [13]. Additionally, the measurement of RNA copies is not easily comparable with PFU/ml values, which have been utilized to determine virus titers in most studies performed over the last 50 years.

Visualization of intracellular LCMV-NP expression by flow cytometry was originally developed by us and others as a tool to assess the viral burden among defined primary cell populations [14], [15], [16] and various cell lines [17], [18], [19] following LCMV infection *in vivo* and *in vitro*, respectively. We have now adapted this methodology to develop a diagnostic approach that accurately quantifies titers of infectious LCMV, provides both enhanced sensitivity and expedience, and can be employed effectively as a surrogate method for analyzing infected tissue samples and viral stocks routinely measured by the plaque assay.

## Results

### Optimization of conditions for detection of LCMV-NP expression by FACS

**Figure 1:**
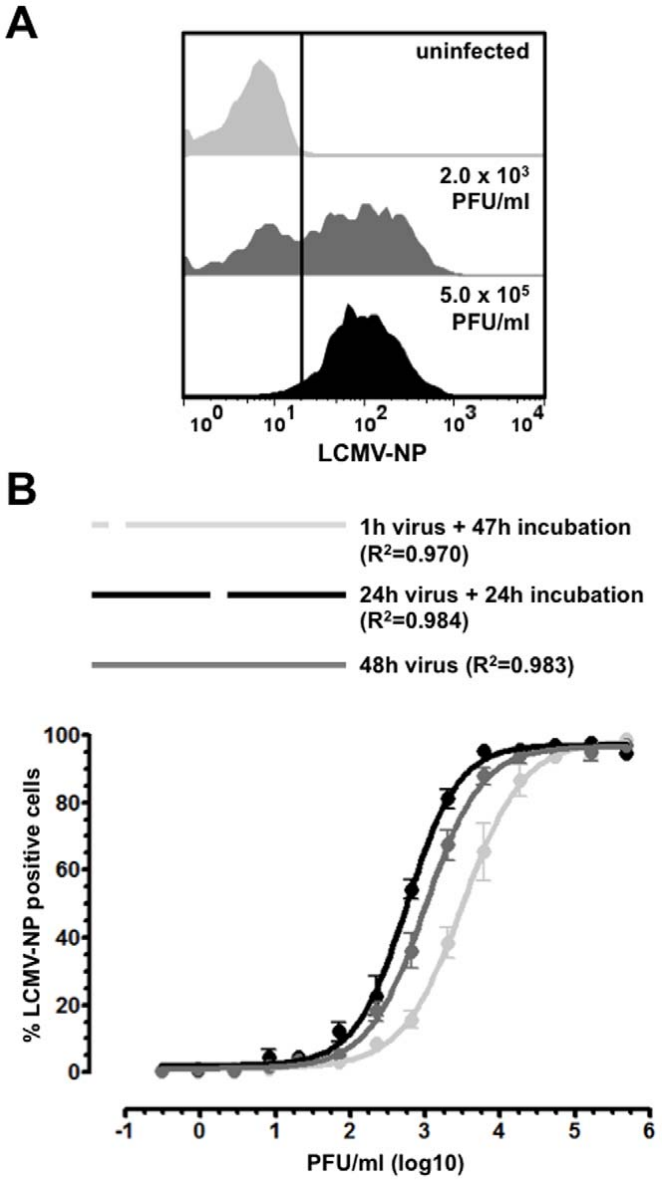
Development and optimization of the LCMV-NP FACS assay. A 3-fold dilution series of a 1×10^6^ PFU/ml LCMV Arm virus stock was produced, incubated with Vero cells, and intracellular LCMV-NP levels were detected by FACS as described in Methods. (**A**) 48 hour virus exposure and FACS detection of LCMV-NP with the unconjugated antibody and a fluorescently conjugated goat anti-mouse secondary antibody. Shown are representative histograms for uninfected, 2×10^3^ PFU/ml and 5×10^5^ PFU/ml LCMV Arm. (**B**) Standard curves were produced from a 3-fold dilution series of LCMV Arm, where virus was incubated with Vero cells for 1 hour, 24 hours, or 48 hours with a total incubation time of 48 hours. Virus titers are represented in PFU/ml. All experiments were performed in duplicate and curves represent compilation of data for at least 3 individual experiments.

In order to be useful experimentally and allow for direct comparison with previous studies, an effective LCMV viral detection method must produce a determinant of viral titers that can be directly related to PFU/ml, the standard measure for virus concentration utilized in the field for more than 50 years. For the LCMV-NP FACS assay, this was achieved by performing a dilution series consisting of between 10 and 13 three-fold dilutions of a 1×10^6^ PFU/ml LCMV Arm virus stock. The corresponding percentages of LCMV-NP positive cells were determined by FACS analysis. Representative histograms illustrating typical LCMV-NP staining for uninfected, high PFU/ml and mid-range PFU/ml amounts of LCMV Arm are shown in ***Figure 1A***. Optimal incubation conditions were determined by varying the length of exposure of Vero cells to virus within the context of a 48 hour overall incubation time, which has been demonstrated in previous experiments (data not shown) to produce a reliable standard curve for LCMV-NP FACS analysis. All conditions tested resulted in standard curves with R^2^ values of at least 0.97 (***Figure 1B***). However, there was a 3-fold increase in sensitivity over the 1 hour virus exposure when the virus was incubated with Vero cells for the entire 48 hours (***Figure 1B***). Incubation of virus for 48 hours produced similar sensitivity to the 24 hour incubation and resulted in a 19-fold increase in sensitivity over the plaque assay (***Table 1***). Therefore, 48 hour virus exposure was chosen as the standard assay condition and was utilized for subsequent validation of LCMV-NP FACS detection with serum samples and organ lysates from LCMV Clone 13 (cl13) and LCMV Armstrong (Arm) infected mice.

**Table 1:** Detection limits and sensitivity for LCMV-NP FACS and Plaque assays.

Method	ID_50_	LOD^1^	PLOQ^2^	Sensitivity^3^
144 hr Plaque Assay	2700 PFU/ml	50 PFU/ml	200 PFU/ml	n/a
48 hr FACS Assay	142 PFU/ml	15 PFU/ml	62 PFU/ml	19
72 hr FACS Assay	35 PFU/ml	1 PFU/ml	2 PFU/ml	77

1 The limit of detection (LOD) equals +/−3× the SD of the mean for uninfected samples.

2 The practical limit of quanitification (PLOQ) equals +/−10× the SD of the mean for uninfected samples.

3 Values represent fold enhanced sensitivity in comparison to the plaque assay.

**Figure 2:**
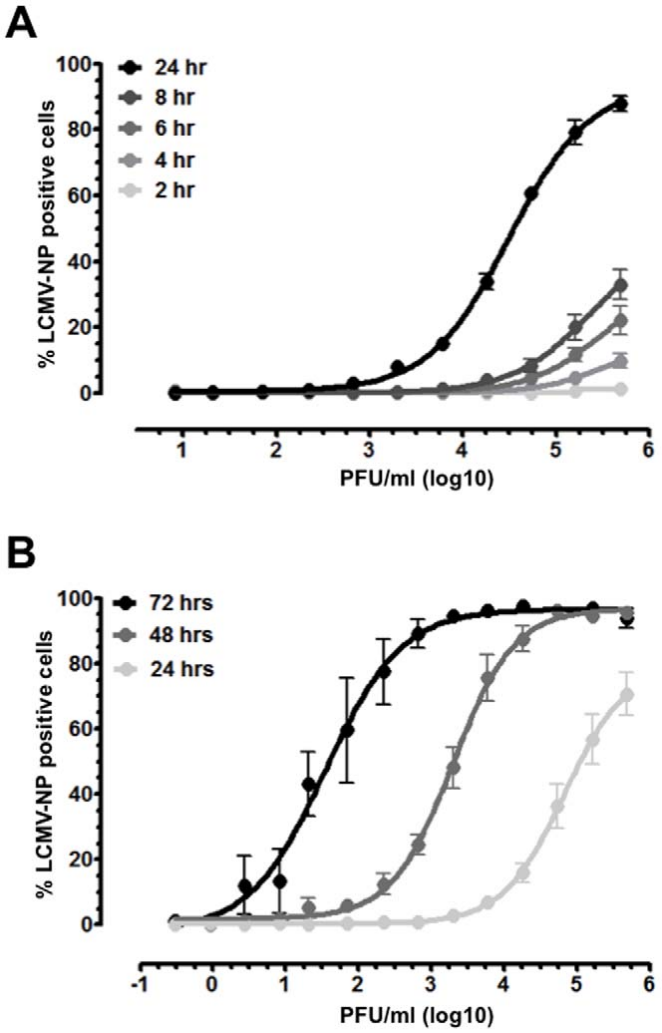
Further optimization of the LCMV-NP FACS assay. (**A**) The minimum virus exposure time required to detect LCMV-NP was ascertained by incubating 3-fold serial dilutions of a 1×10^6^ PFU/ml LCMV Arm stock with Vero cells for 2, 4, 6, 8, and 24 hours and subsequent determination of LCMV-NP expression by FACS analysis. (**B**) The effect of prolonged incubation of virus with Vero cells on LCMV-NP FACS assay sensitivity was determined by extending the 48 hour incubation of 1×10^6^ PFU/ml LCMV Arm stock serial dilutions by 24 hour increments; representative curves for 24, 48 and 72 hour incubations are shown. Virus titers are represented in PFU/ml. All experiments were performed in duplicate and curves represent compilation of data for at least 3 individual experiments.

To determine if the overall assay length could be further diminished, it was essential to establish the minimum time required for detection of intracellular NP during *in vitro* infection of Vero cells. This was achieved by incubation of the above-described dilution series of stock virus with Vero cells for 2, 4, 6, 8, and 24 hours, followed by intracellular detection of LCMV-NP by FACS. At greater than 1.5×10^4^ PFU/ml and 3×10^2^ PFU/ml, LCMV-NP could be detected in Vero cells at 8 hours and 24 hours, respectively (***Figure 2A***). However, even at the highest virus concentration for 24 hour incubation, 100% infection was not achieved. Therefore, incubation of Vero cells with virus for more than 24 hours is required to produce a reliable standard curve that would allow for analysis of unknown samples by LCMV-NP FACS.

Since incubation of LCMV Arm with Vero cells for 48 hours resulted in a reproducible standard curve with enhanced sensitivity over the plaque assay, we tested whether increasing the incubation time could further increase the sensitivity of the LCMV-NP FACS assay. To achieve this, Vero cells were incubated with virus for 72, 96, 120, and 144 hours. Exposure of Vero cells to virus for 72 hours resulted in an additional 4-fold increase in assay sensitivity when compared to 48 hour exposure and a 77-fold enhancement of sensitivity over the plaque assay (***Figure 2B*** and ***Table 1***). There was, however, a decrease in the R^2^ value of the curve at 72 hours due to increased variability in replicate points in the linear range and at lower PFU/ml values. Incubation with virus for 96, 120, and 144 hours (data not shown) further enhanced this variability and also produced an all or none detection scenario. Incubation of Vero cells for 96 hrs or greater with virus resulted in 100% LCMV-NP positive cells in all dilutions containing more than 8 PFU/ml. However, for all dilutions less than 3 PFU/ml, values were below the limit of detection (LOD) threshold (less than 2% LCMV-NP positive cells; data not shown). This abrupt change from complete infection to no infection in consecutive dilutions is likely due to additional viral replication and transmission which occurs during the longer incubation time *in vitro*, as well as the limiting number of infectious virions (in relation to defective interfering virions) that are contained in dilutions with lower PFU/ml values. These results suggest that an additional 24 hours of incubation (72 hours total) could be utilized to enhance sensitivity for detection of samples containing low levels of virus, but that potential assay variability should be taken into account when utilizing standard curves to determine experimental values in these instances. Incubation of virus with Vero cells for more than 72 hours is not recommended for detection purposes due to increased replicate variability and a severe decrease in the linear range that can be utilized for quantification.

### Verification of infectious virus detection

**Figure 3:**
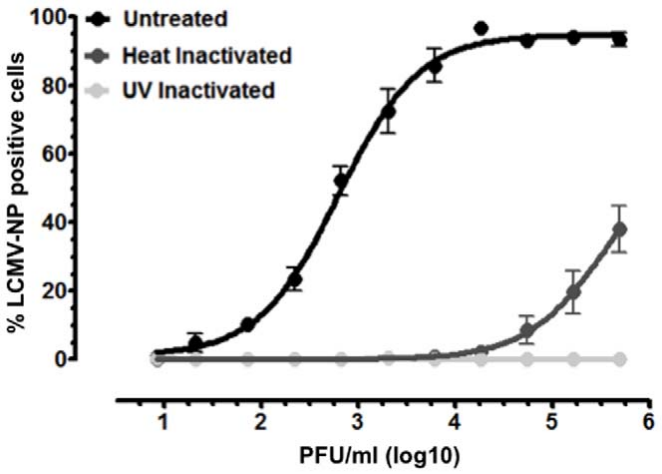
LCMV-NP FACS analysis of inactivated virus. A 1×10^6^ PFU/ml stock of LCMV Arm was inactivated either by exposure to 1 Joule UV irradiation or by incubation at 57°C for 45 minutes. 3-fold serial dilutions were produced for inactivated samples as well as untreated LCMV Arm, incubated with Vero cells for 48 hours, and the percentage LCMV-NP positive cells was determined. Virus titers are represented in PFU/ml. All experiments were performed in duplicate and curves represent compilation of data for at least 3 individual experiments.

In order to ensure that LCMV-NP FACS analysis only detects infectious virus, 1×10^6^ PFU/ml LCMV Arm stocks were inactivated by either exposure to 1 Joule of UV irradiation or incubation at 57°C for 45 minutes. Then, a 3-fold dilution series was produced, incubated with Vero cells for 48 hours, and intracellular staining of LCMV-NP was assessed. Unmanipulated virus was run in parallel as a comparative positive control (***Figure 3***). UV irradiation completely ablated detection of LCMV-NP, even at 1×10^6^ PFU/ml of LCMV Arm, while heat inactivation reduced the percent of LCMV-NP positive cells at the highest viral dose by more than 2 fold, but did not completely inactivate virus, which could be detected in virus concentrations as low as 5×10^4^ PFU/ml (***Figure 3***). By plaque assay, the LOD for heat-inactivated virus was found to be 1×10^4^ PFU/ml (data not shown), which was similar to the value determined by LCMV-NP FACS analysis. These findings confirm our supposition that LCMV-NP FACS analysis only detects infectious virus and indicate that effective heat inactivation protocols likely require extended incubation time or increased temperature to completely ablate the infectious capacity of LCMV.

### Comparative detection limits for plaque and LCMV-NP FACS assays

The Infectivity Dose 50 (ID_50_) values, which were derived from non-linear curve fit analysis and illustrate the PFU/ml value required to achieve infection in 50 percent of Vero cells, were found to be 142 PFU/ml and 35 PFU/ml for the 48 hour and 72 hour LCMV-NP FACS assay, respectively. Using the AF647-conjugated antibody in the 48 hour assay, the ID_50_ was similar to the unconjugated antibody at 217 PFU/ml. The ID_50_ for the plaque assay was determined to be 2700 PFU/ml (***Table 1***) using a 2-fold dilution series of LCMV Arm stock virus (data not shown) and performing non-linear regression analysis on the values determined by plaque assay as detailed in Methods. All sensitivity calculations were determined by comparing the ID_50_ values for each assay rather than the lower LOD because the ID_50_ falls in the linear range of the assay.

Using the 2-fold LCMV Arm dilution series described above and determining the highest dilution that yielded at least one plaque, the LOD for the plaque assay was found to be as low as 12 PFU/ml. In a similar experiment, involving a 3-fold dilution series of the stock virus described above for LCMV-NP FACS, the LOD was 50 PFU/ml (***Table 1***). In practical terms, since there is often a limited volume of serum samples and organ lysates available in comparison to the 500 µl volume required for the plaque assay, 100-fold dilutions are often the lowest utilized. Under these technical restrictions, the practical LOD is 200 PFU/ml, which is one plaque in a 1∶100 dilution of the sample. Overall, it is difficult to determine viral titers based on the presence of one plaque considering the technical nature of the assay and that areas where cells have detached from the plate often resemble plaques.

The lower detection limits for the LCMV-NP FACS assay using either the unconjugated and conjugated LCMV-NP antibodies were calculated as 3 times the standard deviation of the mean for uninfected samples and were found to be equivalent to PFU/ml values that resulted in 2 percent and 1 percent LCMV-NP positive cells, respectively. For the 48 hour LCMV-NP FACS assay with the unconjugated antibody, the 48 hour LCMV-NP FACS assay with the conjugated antibody, and the 72 hour LCMV-NP FACS assay, this value was found to be 15 PFU/ml, 2 PFU/ml, and 1 PFU/ml (***Table 1***). Since there is still approximately a 1% probability at this lower limit that resulting values could fall below the measurements for uninfected samples leading to false negatives, the practical limit of quantification (PLOQ) for the LCMV-NP FACS assay was also determined. This was defined as 10 times the standard deviation of the mean for uninfected samples and corresponded to either 1.5 or 5 percent of LCMV-NP positive cells, which for the 48 hour LCMV-NP FACS with the unconjugated antibody was 62 PFU/ml, the 48 hour LCMV-NP FACS assay with the conjugated antibody was 5 PFU/ml, and the 72 hr FACS assay was 2 PFU/ml (***Table 1***).

### Quantification of viral titers in serum from LCMV cl13 infected mice

**Figure 4:**
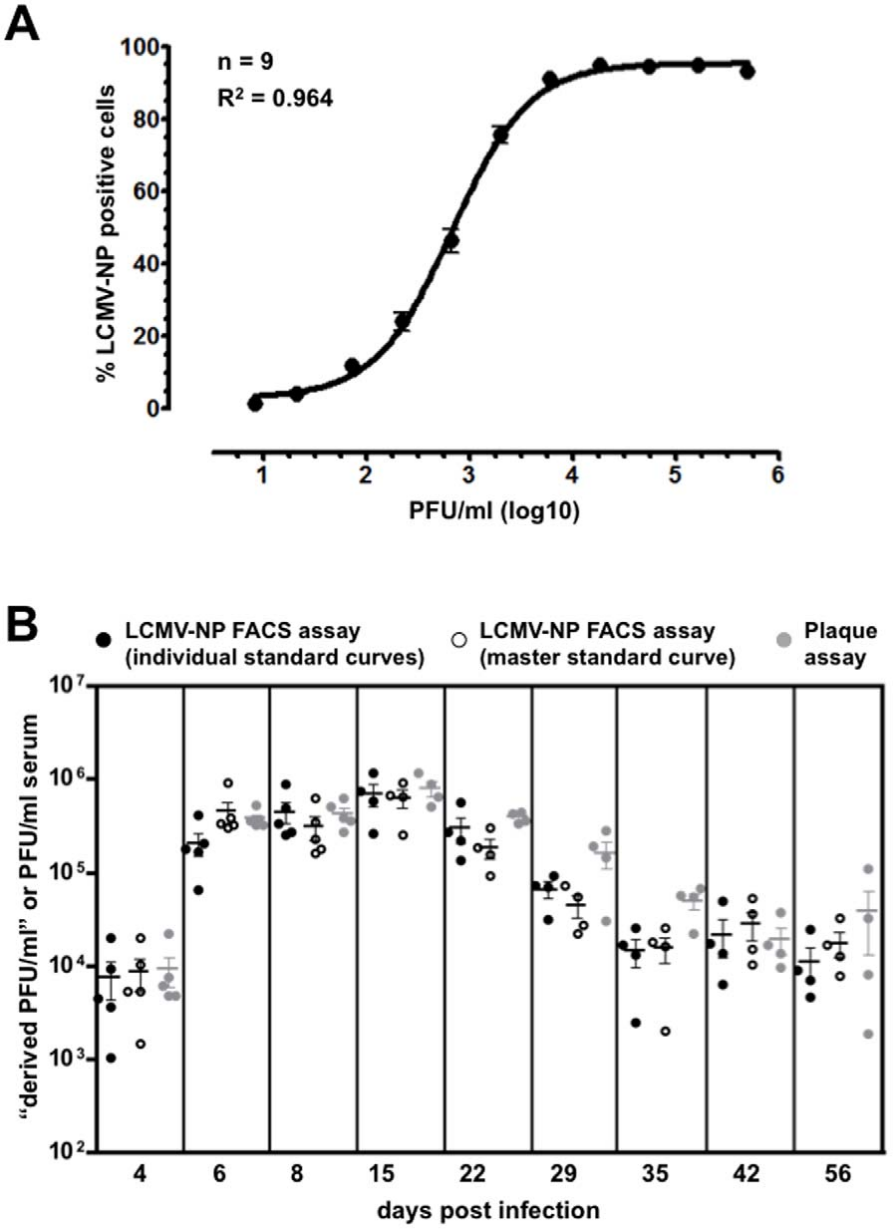
Detection of viral titers in sera of LCMV cl13 infected mice: comparison of LCMV-NP FACS and plaque assays. (**A**) A master standard curve was produced by combining 9 individual standard curves generated for separate experiments by incubation of 10 3-fold serial dilutions of the 1×10^6^ PFU/ml LCMV-Arm virus stock with Vero cells for 48 hours and subsequent determination of LCMV-NP expression by FACS. (**B**) C57/BL6 mice were infected i.v. with 2×10^6^ PFU LCMV cl13, serum samples were collected on 4, 6, 8, 15, 22, 35, 42 and 56 d.p.i., and viral titers were determined by both the LCMV-NP FACS assay (“derived PFU/ml”) and standard plaque assay (PFU/ml). “Derived PFU/ml” values were calculated with individual standard curves generated at the time of experiment (closed symbols) or the above master standard curve (open symbols) generated after completion of all experiments. The symbols represent 5 individual mice for 4, 6, 8 d.p.i. and 4 mice for 15, 22, 29, 35, 42 and 56 d.p.i.

In order to ascertain whether detection of LCMV-NP could be utilized as a suitable replacement for the plaque assay, both methods were employed in parallel to determine PFU/ml values in serum samples from C57/BL6 mice infected with 2×10^6^ PFU LCMV cl13. For the LCMV-NP FACS assay, dilution series of individual serum samples were prepared (6 10-fold dilutions starting at 1∶10), a standard curve containing 10 3-fold dilutions of a 1×10^6^ PFU/ml stock of LCMV Arm was created at the same time, and data were generated and analyzed as detailed in Materials and Methods to calculate individual serum PFU/ml values. For the plaque assay, serum samples were tested in 3 10-fold dilutions starting at 1∶100, the lowest dilution possible due to the small volume of serum obtained from blood (∼70 µl total for each mouse). To ascertain the utility of a “master” rather than individual standard curves, we combined the individual standard curves generated for the 4, 6, 8, 15, 22, 29, 35, 42, and 56 d.p.i. experiments (***Figure 4A***), recalculated the PFU/ml values for individual samples using this “master standard curve”, and found that virus titers determined using individual or master standard curves were not significantly different (***Figure 4B***). Therefore, a master standard curve produced from a known virus stock can be utilized to accurately determine viral titers even if the samples are analyzed on different days, and thus can reduce work load, reagents and materials.

Importantly, for all time points analyzed, the virus titers determined by plaque assay or LCMV-NP FACS analysis were similar (***Figure 4B***) and serum PFU/ml values for LCMV cl13 infected mice that were determined by either assay were indeed comparable to previously reported values [20]. For the LCMV-NP FACS assay, since the values we determined for unknown samples relate directly to the percentage of LCMV-NP positive cells and were derived from known PFU/ml values for a particular LCMV Arm virus stock, we propose to designate them as “derived PFU/ml”. In addition, viral titers for LCMV Arm and LCMV cl13 viral stocks analyzed by both plaque assay and LCMV-NP FACS showed no significant differences (data not shown). These results suggest that analysis of viral titers in both serum samples and viral stocks by the LCMV-NP FACS assay could be confidently utilized as an alternative method to the standard plaque assay.

### Technical constraints of the LCMV-NP FACS assay and the use of unconjugated *vs.* conjugated LCMV-NP antibodies

Analysis of virus in serum and organ samples from infected mice using the LCMV-NP FACS assay, especially with the unconjugated antibody, must take into account a few technical considerations that did not arise when optimization was performed on LCMV Arm virus stocks. At later stages in the course of LCMV cl13 infection (>56 d.p.i.), the LCMV-NP FACS analysis of both serum samples (1∶10 dilution) and tissue homogenates produced high background staining that was also observed in samples stained only with the fluorescent anti-mouse IgG secondary antibody (***Figure S1A/B***, top panels). Since the “background staining” decreased with each dilution of the serum or tissue lysate (data not shown) and was not observed in samples obtained early after infection (***Figure S1A/B***, middle panels), it conceivably resulted from the presence of endogenously generated LCMV-specific antibodies that develop at later time points during the LCMV cl13 infection [2] or otherwise from the non-specific binding of the secondary antibody to compounds present in late-stage sera and organ lysates. In order to overcome these practical and technical constraints, serum samples and organ lysates from LCMV cl13 infected mice (98 d.p.i.) were analyzed with the directly AF647-conjugated LCMV-NP antibody (***Figure S1A/B***, bottom panels). Our results not only demonstrated a nearly complete loss of “background staining” but further indicated that samples with low viral titers not detectable by standard plaque assay in fact could harbor virus as detected by the LCMV-NP FACS assay.

### Increased sensitivity of the LCMV-NP FACS assay: practical application to *in vivo* models of LCMV cl13 and LCMV Arm infection

**Figure 5:**
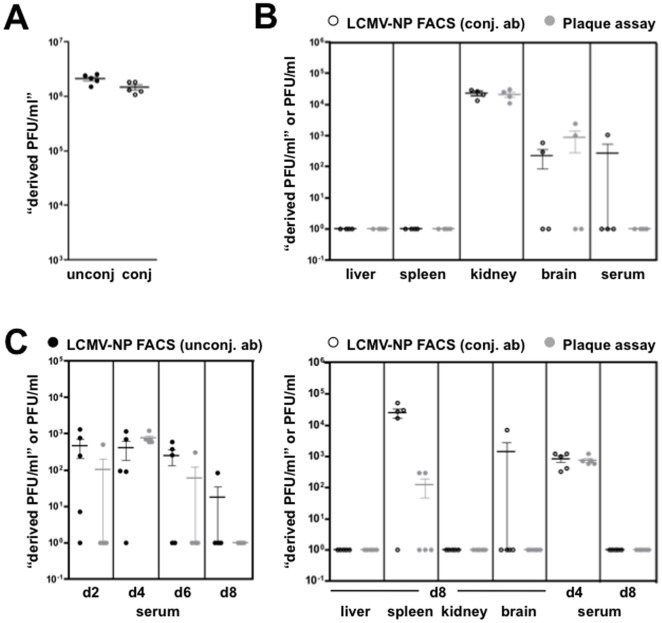
Detection of infectious LCMV under experimental conditions with limited virus titers: comparison of 1-step and 2-step LCMV-NP FACS and plaque assays. (**A**) For validation of the AF647-conjugated LCMV-NP antibody, serum titers of LCMV cl13 infected mice (d.p.i) were analyzed by LCMV-NP FACS using both conjugated and unconjugated versions of the antibody; viral titers are represented in “derived PFU/ml”. (**B**) C57/BL6 mice were infected i.v. with 2×10^6^ PFU LCMV cl13, serum samples were collected and organ lysates prepared at 98 d.p.i., and viral titers were determined in parallel by LCMV-NP FACS using the AF647-conjugated LCMV-NP and standard plaque assay. Virus titers are represented in “derived PFU/ml” (FACS assay) or PFU/ml (plaque assay). (**C**) C57/BL6 mice were infected i.p. with 2×10^5^ PFU of LCMV Arm, serum samples were collected on 2, 4, 6, 8 d.p.i and organ lysates from kidney, spleen, liver and brain were produced on 8 d.p.i. Viral titers were determined for serum 2, 4, 6, 8 d.p.i. by both the LCMV-NP FACS using unconjugated anti-LCMV-NP, and standard plaque assay (left panel). Viral titers were determined on 4 and 8 d.p.i. in serum and in kidney, spleen, liver, and brain from 8 d.p.i. by both the LCMV-NP FACS using the AF647-conjugated anti-LCMV-NP and the standard plaque assay (right panel). Viral titers are represented as in panel B.

In light of the above considerations, we sought to employ the LCMV-NP FACS assay in experimental scenarios where infectious virus is known to be present at very low levels and not reliably detectable by standard plaque assay, *i.e*. in the later stages of an LCMV cl13 infection (>70 d.p.i.) or during the tail end of a primary LCMV Arm infection. To this end, we first established optimal assay conditions for use of the directly AF647-conjugated LCMV-NP antibody (not shown) and demonstrated that parallel assays conducted with both unconjugated and conjugated LCMV-NP antibodies on 8 d.p.i. serum samples obtained from LCMV cl13 infected mice produced equivalent results (***Figure 5A***). We then employed the conjugated LCMV-NP antibody to determine viral titers in 98 d.p.i. serum samples and organ lysates from LCMV cl13 infected mice (***Figure 5B***). Previous studies using the standard plaque assay demonstrated effective clearance of LCMV cl13 from multiple tissues including serum, spleen, and liver by 70–80 d.p.i. but maintenance of elevated LCMV titers in brain and kidney [20]. Our own plaque assay-based quantification of LCMV titers in tissue samples obtained from LCMV cl13 infected mice at 98 d.p.i. confirmed these observations by demonstrating absence of detectable virus in serum, spleen and liver as well as virus persistence in kidney (5/5 mice) and brain (2/5 mice) (***Figure 5B***). In a direct comparison, the LCMV-NP FACS yielded identical results for liver, spleen, brain and kidney (***Figure 5B***). In fact, the brain lysates that tested positive for virus by both plaque assay and LCMV-NP FACS were from the same individual mice (data not shown). These results further confirm that detection by LCMV-NP by flow cytometry can be used as a surrogate method for the plaque assay. Interestingly, we noted detection of infectious virus in a serum sample from one mouse that was deemed negative by plaque assay (***Figure 5B***), suggesting that the LCMV-NP FACS assay might provide information about infection and viral persistence that is beyond the reach of the standard plaque assay.

To explore the possibility for enhanced detection of infectious virus by means of the LCMV-NP FACS assay, we quantified virus titers in the course of a primary LCMV Arm infection (***Figure 5C***), a model of acute viral infection and disease that is associated with early, brief and mostly lower spikes of LCMV titers [20]. Since LCMV Arm is cleared from most tissues by 8 d.p.i. [20], endogenously generated LCMV-specific antibodies would not be expected to interfere significantly with the use of the 2-step LCMV-NP assay, an approach that can provide certain advantages due to its capacity for signal amplification. In 2, 4, 6, 8 d.p.i serum samples from LCMV-arm infected mice, analysis by LCMV-NP FACS using the unconjugated LCMV-NP antibody, for all time points, except 4 d.p.i, resulted in detection of virus in samples from mice that were determined to be negative by plaque assay (***Figure 5C***, left panel). In only one instance was there detection of virus in a sample (from 4 d.p.i. serum) by plaque assay that was determined to be negative by LCMV-NP FACS. It should be noted, however, that analysis of the very same sample with the AF647-conjugated LCMV-NP antibody readily permitted detection of virus (described below and in ***Figure 5C***, right panel). Our results demonstrate that LCMV-NP FACS, using either the conjugated or unconjugated antibody, can detect virus where the standard plaque assay could not, and therefore provides simple analytical methodology for improved quantification of infectious LCMV titers.

When extending the above analyses to various tissues from LCMV Arm infected mice, however, we encountered technical difficulties similar to those described above for the use of unconjugated LCMV-NP antibody with organ lysates obtained from LCMV cl13 infected mice, and which we attribute mostly to background caused by non-specific binding to the large amount of protein and other macromolecules in the tissue homogenates. Again, analysis of both serum and organ lysates by use of the AF647-conjugated LCMV-NP antibody eliminated confounding background staining and allowed for detection of virus in organ lysates prepared from LCMV Arm infected mice at 8 d.p.i. (***Figure 5C***, right panel). In particular, the FACS assay led to detection of virus in spleen and brain lysates that were negative by plaque assay. However, liver and kidney lysates were negative for virus using either method. Viral titers in sera on 4 and 8 d.p.i. determined by the plaque assay and LCMV-NP FACS were nearly identical (***Figure 5C***, right panel) and similar to that determined by LCMV-NP FACS using the unconjugated antibody (***Figure 5C***, left panel). The main limitation of both LCMV-NP FACS and the plaque assay in detecting extremely low levels of virus is the requirement for dilution of serum and organ lysates for analysis and may explain why there is not detection of virus in more samples by LCMV-NP FACS, even with 19-fold increased sensitivity. Another possibility is that these samples simply do not contain infectious virus. Overall, our findings demonstrate that detection of virus by LCMV-NP FACS, especially using the conjugated antibody to avoid background due to non-specific binding or the presence of endogenous LCMV-specific antibodies, constitutes a simple, fast and accurate methodology for the quantification of infectious LCMV across a wide range of viral titers including low amounts of virus that cannot be detected by standard plaque assay.

## Discussion

Recently, several studies illustrating improved methods for detecting LCMV, especially real time RT-PCR for viral RNA, have been reported [9], [10], [11]. It has been shown that RNA detection is a rapid, sensitive method for identifying LCMV viral presence in murine tissues and clinical samples, which has several advantages over the standard plaque assay (***Table 2***). There are several factors that limit the value of RNA analysis for viral quantification in research as opposed to clinical usage. Quantification is generally expressed in RNA copies and only one study [11] represented RNA copies and LOD values in terms of PFU/ml, the standard experimental measure for LCMV and most other infections. Additionally, especially for lower limits of detection, the presence of viral RNA does not necessarily correlate with active viral infection. Real-time RT-PCR is also less cost effective than plaque or FACS-based assays (***Table 2***). Therefore a more sensitive, quicker detection assay that can be used as a surrogate for the plaque assay in detecting infectious virus has many practical and technical advantages over RNA detection for everyday experimental use.

**Table 2:** Comparison of detection methods for LCMV viral titers.

Method	Duration	Objectivity	LOD^1^	Infectious Virus?	Cost
Plaque Assay	6 days	+	50 PFU/ml	+	$
48 hr FACS Assay	2 days	++	15 PFU/ml	+	$$
72 hr FACS Assay	3 days	++	1 PFU/ml	+	$$
RT-PCR Assay	4–5 hours	++	10 PFU/ml	−	$$$

1 The limit of detection (LOD) equals +/−3× the SD of the mean for uninfected samples.

In this study, we describe the development and optimization of a FACS-based assay for quantification of LCMV viral titers which provides several advantages over the commonly utilized plaque assay. These include enhanced sensitivity, decreased assay length, reduced sample volume due to usage of the 96-well plate format, and improved sample processing (8–12 duplicate samples per plate). The 96-well plate format also provides a convenient foundation for high throughput flow cytometry using a variety of fully automated and integrated acquisition and analysis platforms. Overall, assay conditions optimized for a length of 48 hours demonstrated an up to 19-fold enhancement in sensitivity over the standard plaque assay and an increase of incubation time to 72 hours improved the assay sensitivity by a factor of ∼80 in comparison to the 6 day plaque assay. Our application of this methodology to models of acute (LCMV Arm) and chronic (LCMV cl13) viral infection produced comparable results in relation to the standard plaque assay and, under experimental conditions associated with limiting amounts of infectious LCMV, permitted improved virus detection due to enhanced assay sensitivity. Potential drawbacks of the LCMV-NP FACS assay include enhanced sample processing time as compared to the plaque assay as well as the requirement to perform somewhat extended dilution series (usually 4–6 dilutions) to assure that the experimental readout (% LCMV-NP positive cells) falls within the linear range of the corresponding standard curve, a critical condition to permit the accurate calculation of PFU/ml values. In situations where results are not needed immediately, only a general estimate of the virus titer is necessary, or the titer of new viral stocks is being measured, the traditional plaque assay might therefore be a more practical option. However, since the exact quantification of LCMV titers often constitutes a critical experimental readout [16], the LCMV-NP FACS assay can provide a faster, more sensitive and accurate methodological alternative in a multitude of experimental and naturally occurring settings. Lastly, this assay can, in principle, be adapted to other viruses and model systems (eg, influenza) where rapid, objective, enhanced and accurate quantification of virus titers is of diagnostic importance.

## Materials and Methods

### LCMV-NP antibody production and fluorescent conjugation

The primary mouse IgG2a antibody specific for the LCMV-NP was produced from the M-Se 113 Hybridoma (generously provided by Dr. M. Oldstone) and was purified by the University of Colorado Cancer Center Protein Production, Monoclonal Antibody, and Tissue Culture Shared Resource using standardized protocols. In brief, the hybridoma was seeded at a low density of 5.0×10^5^ cells/ml in RPMI with 7% fetal calf serum (FCS), 1% penicillin, 1% streptomycin, and 1% L-glutamine, and expanded in spinner vessels (BellCo Glass, Vineland, NJ) for 7 days until growth reached the log phase. The supernatant was harvested and run twice over a 1 ml Hi-Trap Protein G Column (GE Healthcare, Piscataway, NJ), washed with PBS, and eluted using a low pH elution glycine buffer (Perkin-Elmer, Waltham, MA). The elutant was dialyzed two times in 1 L of PBS for 3 hours, then analyzed for purity and protein concentration by Coomassie Blue Gel (Bio-Rad, Hercules, CA). The purified antibody was stored at 2 mg/ml in 500 µl aliquots at −20°C until use for FACS detection utilizing a Cy5-, DyLight 649- or Alexa Fluor 647 (AF647)-conjugated goat anti-mouse IgG F(ab′)_2_ fragment as a secondary antibody (Jackson Immunoresearch Laboratories, Inc., West Grove, PA). Functional activity and optimal antibody concentration for detection of LCMV were determined by FACS analysis using Vero cells infected with 1.0×10^6^ PFU/ml LCMV Armstrong (Arm) for 1 hour followed by media change and additional 48 hours of incubation at 37°C, 5% CO_2_ as described [19]. Uninfected cells stained with both primary and secondary antibodies and virus-infected cells stained with the secondary antibody alone were utilized as negative controls. Alternately, the LCMV-NP antibody was fluorescently conjugated using an AF647 Protein Labeling Kit (Molecular Probes, Carlsbad, CA).

### Virus production, storage and inactivation

The LCMV Armstrong (Arm) and LCMV clone 13 (cl13) strains [21] utilized in all experiments were originally obtained from Dr. M. Oldstone, and stocks were produced as described previously [22]. Viral titers for stock virus were determined using the standard plaque assay (described below). Long-term LCMV Arm and cl13 stocks utilized for infections were stored in 1 ml aliquots in liquid nitrogen and thawed immediately before use. Working stocks for LCMV Arm utilized for the standard curve and assay optimization were diluted from liquid nitrogen stocks to 1×10^6^ PFU/ml and stored in 1 ml aliquots at −80°C until use. LCMV Arm was inactivated using both heat and UV techniques following previously published methods [23], [24]. For UV inactivation, 1×10^6^ PFU/ml stocks of LCMV Arm were subjected to 1 Joule/cm^2^ of UV irradiation in a Spectrolinker XL-1500 UV cross-linker (Spectronics Corporation, Westbury, NY). Heat inactivation was achieved by incubating 1×10^6^ PFU/ml stocks of LCMV Arm in a 57°C water bath for 45 minutes. All inactivation procedures were performed immediately prior to addition of virus to Vero cells.

### LCMV plaque assay

LCMV plaque assays were performed as described previously [25], [26]. Vero cells (originally obtained from Dr. M. Oldstone [26]) were re-suspended in MEM media containing 7% FCS, 1% penicillin, 1% streptomycin, and 1% L-glutamine, plated in 6 well tissue culture plates at 1×10^5^ cells/ml and incubated overnight at 37°C and 5% CO_2_. Vero cells were then incubated with 2-fold dilutions of a 1×10^6^ PFU/ml LCMV Arm virus stock (for determination of assay sensitivity) or 10-fold dilutions of serum samples and organ lysates in a total volume of 500 µl for 1 hour at 37°C and 5% CO_2_. The supernatant was aspirated and replaced with an overlay consisting of a 1∶1 mixture of 1% agarose and EMEM medium containing 14% FCS, 2% L-glutamine, 2% penicillin, and 2% streptomycin. The plates were then incubated at 37°C and 5% CO_2_ for 6 days. On day 6, the agarose overlay was removed, cells were fixed in 25% formalin for 45 minutes, and stained with 1× crystal violet (Sigma, St. Louis, MO). Plaques were visually counted and PFU/ml values were determined using the following formula: # of plaques per well ×2/dilution factor.

### LCMV-NP FACS assay

FACS detection of LCMV-NP was modified from previously described protocols [14], [15], [19], [27]. Vero cells were plated in flat bottom 96-well tissue culture plates in MEM media at 5×10^4^ cells per well and incubated at 37°C and 5% CO_2_ overnight. Serial dilutions of the 1×10^6^ PFU/ml LCMV Arm viral stock (11 to 14 duplicate 3-fold dilutions) were prepared in MEM media and 50 µl of each dilution was added to Vero cells. To avoid variation in detection that could occur as a result of evaporation during longer incubations, an additional 50 µl of MEM media was added to each well for a final volume of 100 µl and Vero cells were incubated with virus for the indicated time period for each individual experiment. Cells were then washed with cold 1× PBS and incubated with 60 µl of warm 0.25% Trypsin-EDTA (Gibco, Carlsbad, CA) at 37°C for 5 minutes. 60 µl of cold MEM media with 7% FCS was then added to each well, the cells were removed by gentle pipetting, and transferred to a 96-well V-bottom plate. Cells were pelleted by centrifugation at 1600 rpm, re-suspended in PFA with 1% saponin, and incubated at room temperature for 15 minutes. The cells were pelleted by centrifugation at 1600 rpm and washed twice in PBS with 1% saponin. The cells were then re-suspended in PBS with 1% saponin containing 4 µg/ml of the primary LCMV-NP mouse IgG2a or 9.75 µg/ml of the AF647-conjugated LCMV-NP antibody and incubated at room temperature for 40 minutes. The cells were pelleted by centrifugation at 1600 rpm and washed twice in PBS with 1% saponin. Next, the cells were re-suspended in PBS with 1% saponin containing 5 µg/ml of Cy5-, DyLight 649- or AF647-conjugated goat anti-mouse F(ab′)_2_ (Jackson Immunoresearch Laboratories, Inc., West Grove, PA) and incubated at room temperature for 30 minutes (for the directly conjugated antibody, this step was omitted). The cells were then washed twice in PBS with 1% saponin, re-suspended in FACS buffer (PBS, 1% FCS and 0.5% sodium azide), and analyzed on the FACSCalibur Flow Cytometer (BD Biosciences, Franklin Lakes, NJ). Virus positive populations were determined by negatively gating on uninfected samples. Gates were adjusted to account for fluctuations in fluorescent background (compared to samples stained with the fluorescent secondary antibody only as a negative control) and variations in fluorescent intensity of negative populations, especially for serum samples and organ lysates from infected mice (see below).

### Mice, infections and quantification of viral titers in serum and organs

Male C57/BL6 mice were infected intravenously (i.v.) with 2×10^6^ PFU LCMV cl13 or intraperitoneally (i.p.) with 2×10^5^ PFU LCMV Arm, blood was collected at various days post infection (d.p.i.) as indicated in ***Figures 4***, ***5*** and ***S1***, and serum was obtained by centrifugation at 8000 rpm for 5 minutes at 4°C. For determination of LCMV titers in solid tissues (spleen, liver, brain, kidney; ***Figures 5*** and ***S1***), harvested organs were weighed, a weight-adjusted volume of MEM media with 7% FCS was added to each tissue, and organ lysates effectively corresponding to a 1∶10 dilution were produced by grinding the organs with a tissue homogenizer. Serum and organ homogenates were diluted in 10-fold increments and 2-day LCMV-NP FACS and 6-day standard plaque assays were performed in parallel as described above. For the LCMV-NP FACS assay, a standard curve was produced using 3-fold dilutions of a 1×10^6^ PFU/ml LCMV Arm stock, and “derived PFU/ml” values for serum samples were interpolated from the standard curve by non-linear regression analysis using Prism 5.0 software (GraphPad Software, Inc., San Diego, CA). Since the accurate derivation of PFU values is contingent on experimental readouts above detection limits (*i.e.* >2% LCMV-NP positive Vero cells) and ideally within the linear range of the standard curve, it is important to consider the exact number of dilutions to be analyzed: in general, the higher the expected virus titers, the more dilutions should be prepared, and in most cases 4–6 10-fold dilutions were sufficient to meet the above criteria (thus permitting the duplicate analysis of 8–12 samples per 96-well plate). For samples with expectedly lower virus titers, we recommend tighter-spaced dilution series (1∶3 to 1∶5). For representative dot plots of dilutions containing low levels of virus that scored positive and negative, respectively, see ***Figure S1A/B***, bottom panels. All procedures were performed in accordance with NIH guidelines, were approved by the University of Colorado Institutional Animal Care and Use Committee (#B-70210(05)1E), and all efforts were made to minimize suffering of animals.

### Statistical analysis and calculation of FACS and plaque assay sensitivity

Statistical analysis was conducted using the Prism 5.0 statistical program (GraphPad Software, Inc., La Jolla, CA). All standard curves were generated and unknown serum and tissue values were interpolated using non-linear curve fit analysis; “derived PFU/ml” corresponding to infection of 50% of Vero cells were designated as the infectivity dose 50 (ID_50_). For the plaque assay, the ID_50_ was determined by non-linear regression analysis from a 2-fold dilution series using the same 1×10^6^ PFU/ml LCMV Arm stock utilized to generate curves for the LCMV-NP FACS assay. Differences in the ID_50_ values were employed to determine the fold-enhanced sensitivity in comparison to the plaque assay displayed in ***Table 1***. The limit of detection (LOD) and practical limit of quantitation (PLOQ) for the LCMV-NP FACS assay using the unconjugated and conjugated antibodies were determined as plus or minus 3 and 10 SD of the mean of “LCMV-NP^+^” cells in uninfected replicates (n>50 and n = 4, respectively).

## Supporting Information

Figure S1 **Technical considerations for detection of virus in serum samples and organ lysates using LCMV-NP FACS.** (**A**) Representative dot plots generated by LCMV-NP FACS analysis of serum samples obtained from LCMV cl13 infected mice 70 d.p.i. (top panel), 8 d.p.i. (middle panel) and 98 d.p.i (bottom panel). Note the enhanced “background staining” of the 2-step procedure (top panel, compare “αLCMV-NP” +II° and “II° only”) and the reduction thereof using the directly conjugated LCMV-NP antibody (bottom panel, “αLCMV-NP-AF647”). (**B**) Representative dot plots of LCMV-NP FACS analyses conducted with 1∶30 dilutions of liver lysates from LCMV cl13 infected mice (top panel: 107 d.p.i., middle panel: 8 d.p.i. and bottom panel: 98 d.p.i.). As above, note the background in the 2-step (top) but not 1-step (bottom) staining procedure.
(TIF)
