# Supplementary figures and images for: Accelerated and Improved Quantification of Lymphocytic Choriomeningitis Virus (LCMV) Titers by Flow Cytometry
Source: PLoS One. 2012 May 17;7(5):e37337. doi: 10.1371/journal.pone.0037337 (PMC3355137; doi:10.1371/journal.pone.0037337)

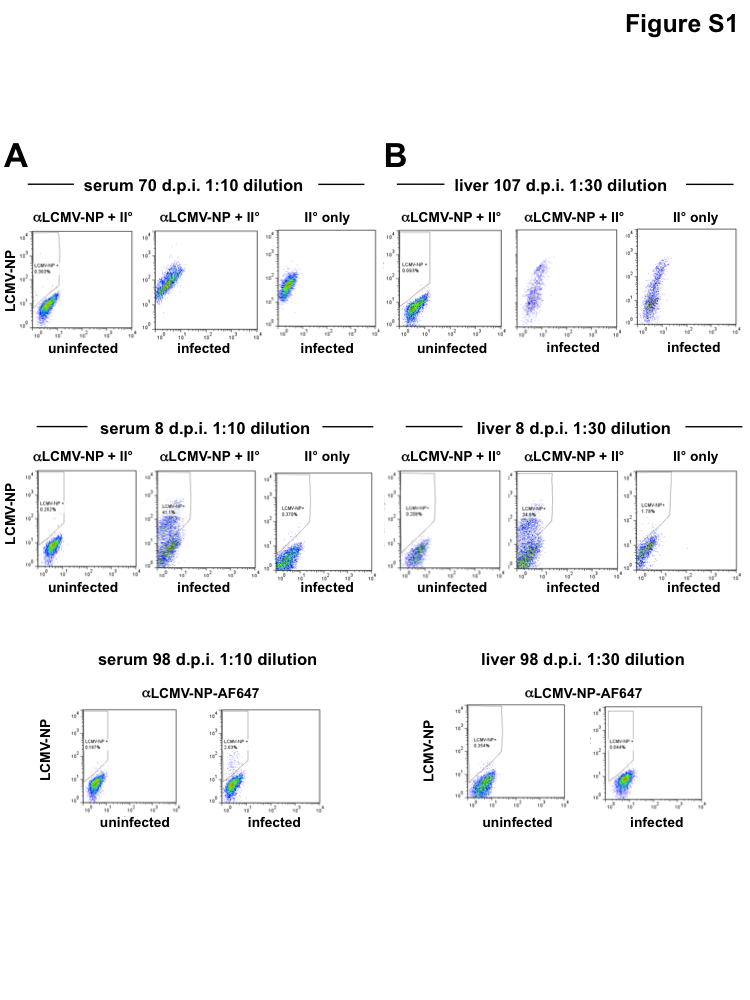

Supplement: Figure S1 — Technical considerations for detection of virus in serum samples and organ lysates using LCMV-NP FACS. (A) Representative dot plots generated by LCMV-NP FACS analysis of serum samples obtained from LCMV cl13 infected mice 70 d.p.i. (top panel), 8 d.p.i. (middle panel) and 98 d.p.i (bottom panel). Note the enhanced “background staining” of the 2-step procedure (top panel, compare “αLCMV-NP” +II° and “II° only”) and the reduction thereof using the directly conjugated LCMV-NP antibody (bottom panel, “αLCMV-NP-AF647”). (B) Representative dot plots of LCMV-NP FACS analyses conducted with 1∶30 dilutions of liver lysates from LCMV cl13 infected mice (top panel: 107 d.p.i., middle panel: 8 d.p.i. and bottom panel: 98 d.p.i.). As above, note the background in the 2-step (top) but not 1-step (bottom) staining procedure. (TIF) [file pone.0037337.s001.tif]
